# Expression, and Molecular and Enzymatic Characterization of Cu-Containing Nitrite Reductase from a Marine Ammonia-Oxidizing Gammaproteobacterium, *Nitrosococcus oceani*

**DOI:** 10.1264/jsme2.ME11310

**Published:** 2012-04-28

**Authors:** Keitaro Kondo, Katsuhiko Yoshimatsu, Taketomo Fujiwara

**Affiliations:** 1 Department of Biological Science, Graduate School of Science, Shizuoka University, 836 Oh-ya, Suruga-ku, Shizuoka 422–8529, Japan

**Keywords:** Cu-containing nitrite reductase, nitrifier denitrification pathway, marine ammonia-oxidizing gammaproteobacteria, *Nitrosococcus oceani*

## Abstract

Ammonia-oxidizing bacteria (AOB) remove intracellular nitrite to prevent its toxicity by a nitrifier denitrification pathway involving two denitrifying enzymes, nitrite reductase and nitric oxide reductase. Here, a Cu-containing nitrite reductase from *Nitrosococcus oceani* strain NS58, a gammaproteobacterial marine AOB, was expressed in *Escherichia coli* and purified to homogeneity. Sequence homology analysis indicated that the nitrite reductase from *N. oceani* was phylogenetically closer to its counterparts from denitrifying bacteria than that of the betaproteobacterium *Nitrosomonas europaea*. The recombinant enzyme was a homotrimer of a 32 kDa subunit molecule. The enzyme was green in the oxidized state with absorption peaks at 455 nm and 575 nm. EPR spectroscopy indicated the presence of type 2 Cu. Molecular activities and the affinity constant for the nitrite were determined to be 1.6×10^3^ s^−1^ and 52 μM, respectively.

Many kinds of microorganisms, including bacteria, archaea, and fungi, can grow anaerobically by denitrification (37). Denitrification is a respiratory process that utilizes nitrate as a terminal electron acceptor instead of oxygen. The biochemistry of denitrification has been well investigated in several denitrifying microorganisms; nitrate is converted to N_2_ through nitrite, nitric oxide (NO) and nitrous oxide (N_2_O) by four successive reduction steps catalyzing dissimilatory nitrate reductase, nitrite reductase, NO reductase and N_2_O reductase, respectively (37). Nitrite reductase catalyzes the reduction of nitrite and produces NO using two types of enzymes with distinct molecular structures, a Cu-containing nitrite reductase and the “cytochrome *cd*_1_” containing hemes *c* and *d*_1_ as the prosthetic cofactors (37). The Cu-containing enzyme that is encoded in the *nirK* gene is distributed widely in denitrifying bacteria, and also in fungi and halophilic archaea with denitrifying abilities (14, 22, 37). Cytochrome *cd*_1_, encoded in the *nirS* gene, is present in denitrifying bacteria and additionally in hyperthermophilic denitrifying archaea (11, 37).

Interestingly, Cu-containing nitrite reductase is reported not only in denitrifying microorganisms but also in non-denitrifying microorganisms. Ammonia-oxidizing bacteria (AOB) are chemoautotrophic microorganisms that gain energy by the oxidation of ammonia to nitrite coupled with aerobic respiration (35). The biochemistry of AOB has been investigated using mainly *Nitrosomonas europaea*, a betaproteobacterial AOB (β-AOB) (2, 35). Ammonia oxidation is carried out through two sequential reactions involving ammonia monooxygenase (AMO): the conversion of ammonia to hydroxylamine (NH_2_OH) using molecular oxygen and the conversion of NH_2_OH by NH_2_OH oxidoreductase (HAO), which catalyzes a four-electron oxidation, to generate nitrite (2, 5). Nitrite reductase has been purified from *N. europaea* as a soluble, blue copper protein (8). The amino acid sequence of the enzyme, encoded in the NE0924 gene in the *N. europaea* genome, is homologous to those of Cu-containing nitrite reductase from denitrifying microorganisms (18). Total genomic analysis also indicated the presence of the NO reductase gene in the *N. europaea* genome (7); however, the genes for dissimilatory nitrate reductase and N_2_O reductase are absent in the genome (7).

*N. europaea* can grow organotrophically under anaerobic conditions with several organic compounds as a substrate and nitrite as the terminal electron acceptor, but the rate of growth by denitrification is very slow (29). Therefore, nitrite reduction to produce N_2_O *via* NO by the two denitrifying enzymes, nitrite reductase and NO reductase, does not participate prominently in an anaerobic energy-generating process in *N. europaea*. Nitrite is the main product of ammonia oxidation in AOB, whereas it is reported that the production of small amounts of NO, N_2_O and N_2_ is associated with ammonia oxidation by AOB cells (26, 30, 31, 36). These gases are also produced when the nitrite is added to intact cells, suggesting that a reduction process similar to microbial denitrification may occur in AOB (27, 30). Due to loss of AMO activity upon exposure to nitrite and to the severe cytotoxicity of NO that is generated by nitrite reduction, the physiological function of nitrite reductase and NO reductase seems to be detoxification of the chemical rather than energy metabolism by denitrification (3, 32).

The putative process for the detoxification of nitrite and NO, which has been called the nitrifier denitrification pathway, is also identified in a gammaproteobacterial AOB (γ-AOB), *Nitrosococcus oceani*, that globally inhabits the marine environment (33, 34). Similar to *N. europaea* and other β-AOBs, *N. oceani* can reduce nitrite and generate N_2_O by nitrifier dentrification (5). The *nirK* gene (noc_0089) and *norCBQD* operon (noc_1850–1847, reverse direction) encoding nitrite reductase and NO reductase, respectively, were found in the genome of *N. oceani* ATCC19707, a type strain of the bacterium (21), although these enzymes have not been purified and their enzymatic characteristics remain unclear.

Recently, we have been studying the biochemistry of ammonia oxidation and its relative processes in a marine γ-AOB, strain NS58, which was isolated in Tokyo Bay and is phylogenetically very close to *N. oceani* ATCC19707 (13). In this study, the Cu-containing nitrite reductase of *N. oceani* NS58 was prepared as a recombinant protein, and its molecular and catalytic properties were analyzed. This is the first report of the kinetic parameters of nitrite reductase, which is involved in the nitrifier denitrification pathway of γ-AOB.

## Materials and Methods

### Cultivation of organism

*N. oceani* NS58 is a marine γ-AOB isolated from coastal marine sediment in Tokyo Bay and was kindly supplied by Dr. H. Urakawa (Florida Gulf Coast Univ.). Medium composition and protocol for large-scale cultivation in 10 L volume three times with *N. oceani* NS58 followed a previous report (13). Genomic DNA of the NS58 was prepared by a standard method.

### Cloning, sequencing, and construction of expression vector

Oligonucleotide primers for PCR amplification of the DNA region encoding the nitrite reductase precursor of *N. oceani* NS58 were designed based on available genome information of *N. oceani* ATCC19707. The forward primer, NcnirKf, was 5′-GCA TAT GAA AAA GTT AAT AAA G-3′ (artificial *Nde*I restriction site is underlined), and the reverse primer, NcnirKr, was 5′-GGTCGACT CAATCTGCATTAATAGG-3′ (*Sal*I site is underlined). Amplification was carried out using KOD-plus DNA polymerase (TOYOBO, Osaka, Japan) and NS58 genomic DNA as a template. The 1,080 bp PCR product obtained was ligated to a pCR-blunt vector (Invitrogen, Carlsbad, CA, USA), yielding pCRNcNirKp. The insert of the plasmid was sequenced using a Li-Cor model 4200 DNA sequencer (Li-Cor, Lincoln, NE, USA). The nucleotide sequence of the PCR product was completely identical to that of the noc_0089 gene of *N. oceani* ATCC19707. PCR was also carried out to amplify the *nirK* gene without the 60-bp nucleotides at its 5′-end that correspond to a putative transmembrane translocation signal sequence using genomic DNA as a template. The forward primer NcnirK^ns^f, 5′-CCATATGGCTGATGGAGAAGCATCATC- 3′ (*Nde*I site is underlined), and reverse primer NcnirKr were used. Using similar PCR protocols, the 1,020-bp product was obtained and inserted into the cloning vector, yielding pCRNcNirKm. Homology search and phylogenetic analysis were performed using Blast and MEGA programs, respectively.

The insert of pCRNcNirKp was digested with both *Nde*I and *Sal*I and then ligated to a pET21a+ expression vector (Novagen; Merck, Darmstadt, Germany) linearized by the same restriction enzymes, yielding expression vector pETNcNirKp. To express the recombinant protein, the pETNcNirKp vector was transduced into *E. coli* BL21(DE3)-CodonPlus (Merck) as an expression host cell. The expression vector for nitrite reductase without a signal sequence in the N-terminal was constructed using the same procedure, and the pETNcNirKm thus yielded was also transduced into BL21 host cells for expression.

### Purification of recombinant nitrite reductase

BL21/pETNcNirKp (or pETNcNirKm) was cultivated in 20 mL of 2×YT medium supplemented with 100 μg mL^−1^ ampicillin at 37°C overnight with shaking at 180 rpm. The overnight culture was inoculated into 2 L of 2×YT/ampicillin induction medium and incubated at 37°C with shaking at 150 rpm. When the optical density of the medium at 600 nm reached 0.6–0.8, an IPTG stock solution (40 mM) was added to the medium to a final concentration of 100 μM for induction of the recombinant protein. After incubation at 25°C with shaking at 150 rpm for 4 h, the cells were collected by centrifugation and stored at −80°C until use.

Pelleted cells of induced BL21/pETNcNirKm were suspended in 40 mL of 10 mM Tris-HCl (pH 8.0) containing 250 mM NaCl and 10 μM phenylmethylsulfonyl fluoride (PMSF) (buffer A). The suspension was sonicated using a VP-30S supersonic oscillator (Taitec, Koshigaya, Japan) for 30×20 s at full power on ice to disrupt cells. The resulting solution was centrifuged at 14,000×*g* for 30 min to precipitate insoluble materials, including inclusion bodies of the recombinant protein and debris. The supernatant that contained the recombinant apoprotein having no nitrite reducing activity was subjected to ammonium sulfate fractionation. Fine granules of ammonium sulfate were carefully added to the supernatant to 30% saturation under continuous stirring on ice. After 1 h, the solution was centrifuged at 10,000×*g* for 10 min to remove the precipitate. The supernatant obtained was further mixed with ammonium sulfate to 50% saturation, then centrifuged again under the same condition. The supernatant was dialyzed against 500 mL of 10 mM Tris-HCl (pH 8.0) containing 10 μM PMSF and 100 μM CuSO_4_ for incorporation of Cu^2+^ ions into the recombinant apoprotein for refolding and activation. The resulting solution that contained recombinant nitrite reductase in the active state was concentrated using an Amicon Ultra Centrifugal Filter Unit 50k (EMD Millipore, Billerica, MA, USA). The concentrated sample was applied to an anion-exchange chromatography column (1×10 cm) of DEAET-oyopearl 650M gel (Tosoh, Tokyo, Japan) that had been equilibrated with 10 mM Tris-HCl (pH 8.0) containing 10 μM PMSF (buffer B). The recombinant enzyme adsorbed on the column was eluted by a salt gradient generated from 100 mL each of buffer B and buffer B containing 0.2 M NaCl. The fractions that showed nitrite reducing activity were collected, and ammonium sulfate was added to 90% saturation. The solution was centrifuged at 22,000×*g* for 30 min. The pelleted material was suspended in 1 mL of buffer A, then applied to a column (2×120 cm) of Sephacryl S-300 (GE Healthcare, Little Chalfont, U.K.) that had been equilibrated with buffer A. The fractions showing nitrite reducing activity were collected and ammonium sulfate was added to 60% saturation. The solution was loaded onto a Sepharose CL-4B (GE Healthcare) column (1×10 cm) that had been equilibrated with buffer A, which was 60% saturated with ammonium sulfate. The recombinant enzyme that adsorbed on the column by hydrogen-bonding interaction between the protein molecules and the Sepharose resin was eluted with a linear gradient of 100 mL each of buffer A 60% saturated with ammonium sulfate and buffer A. The fractions showing enzymatic activity were collected, concentrated by centrifugal filtration, and used as the purified sample for experiments.

### Other experiments

Nitrite reducing activity was determined by measuring the rate of nitrite consumption in the assay solution. A 30 mM wide-range CPT buffer (containing 10 mM each of citric acid, phosphoric acid, and Tris base), of which the pH had been adjusted to a suitable value from 4.0 to 10.0, was used to buffer the pH of the assay solution. The assay solution (0.1 mL in volume) containing 30 mM CPT buffer, 200 μM sodium nitrite, 12.5 μM methylviologen (MV), and the surface of the enzyme preparation in the test tube was overlaid with mineral oil to prevent contact with air. The reaction was started by mixing 5 μL of 10 mg mL^−1^ sodium dithionite as the reductant. The solution was incubated at 37°C for 10 min, and the reaction was stopped by adding 0.9 mL distilled water; then, the concentration of nitrite remaining in the assay solution was determined spectrophotometrically by a diazo-coupling method (25).

Spectroscopic analysis in the visible region was carried out in a 1 cm light-path cuvette using an MPS-2000 spectrophotometer (Shimadzu, Kyoto, Japan). SDS-PAGE was carried out according to the method of Schägger and Jagow (28). The protein concentration was measured using a BCA protein assay kit (Pierce, Rockford, IL, USA) with bovine serum albumin as the standard. Bovine liver catalase (molecular weight: 232,000), alcohol dehydrogenase (150,000), hemoglobin (67,000), carbonic anhydrase (29,000), and horse mitochondrial cytochrome *c* (12,500) were used as standard proteins for determination of the molecular weight of recombinant NirK by Sephacryl S-300 gel filtration. Copper concentrations were determined with a polarized Zeeman atomic absorption spectrophotometer Z-8270 (Hitachi, Tokyo, Japan) after the samples had been dialyzed against 10 mM Tris-HCl buffer, pH 8.0, containing 100 μM EDTA for 2 h. The N-terminal sequences of the purified preparations were analyzed using a PPSQ-21 protein sequencer (Shimadzu). The EPR spectrum of the purified recombinant NirK (9.1 mg mL^−1^) was recorded on a JEOL spectrophotometer model JES-FE2XG (JEOL, Tokyo, Japan). All chemicals used in the experiments were of the highest grade commercially available.

### Nucleotide sequence accession numbers

The sequence data obtained have been assigned accession number FR847063 in the European Molecular Biology Laboratory (EMBL) database.

## Results and Discussion

To purify the nitrite reductase, about 1.5 g (wet weight) of the cell pellet of *N. oceani* NS58 obtained by large-scale cultivation were used as the starting material. According to a previous report on the purification of *N. europaea* nitrite reductase, the enzyme was co-isolated with hydroxylamine oxidoreductase by gel filtration because it is soluble and has a high molecular weight (8, 13); however, in the case of *N. oceani* NS58, no nitrite reducing activity was detected in any fraction obtained by gel filtration. On the other hand, generation of N_2_O from intact cells of *N. oceani* by the nitrifier denitrification pathway has been demonstrated by inhibition analysis (5). These results suggested a low expression level of the enzymes in the nitrifier denitrification pathway in the bacterium.

Therefore, we tried to overexpress the recombinant enzyme in *Escherichia coli* as a host cell which did not express respiratory nitrite reductase in an aerobic condition, and purification and molecular and enzymatic characterization of the protein were carried out. The nitrite reductase precursor, which is encoded by the noc_0089 gene of *N. oceani* ATCC19707, includes a putative Sec-signal sequence in its N-terminal, indicating periplasmic localization of the mature enzyme in the bacterial cell. First, the entire region of the *nirK* gene of the NS58 was amplified by PCR using oligonucleotide primers designed based on the sequence of the noc_0089 gene of the *N. oceani* ATCC19707. The nucleotide sequence of the 1084-bp length product (without the sequence of primers used for PCR) was completely identical to that of *N. oceani* ATCC19707.

The putative amino acid sequence of the nitrite reductase from *N. oceani* was aligned with those from β-AOB (*N. europaea*), denitrifying bacteria (*Alcaligenes faecalis*, *Alcaligenes xylosoxidans*, *Achromobacter cycloclastes* and *Bradyrhizobium japonicum*), denitrifying archaea (*Haloarcula marismortui* and *Haloferax volcanii*), denitrifying fungi (*Fuzarium oxysporum* and *Aspergillus oryzae*), nondenitrifying pathogenic bacteria (*Neisseria gonorrhoeae* and *Burkholderia pseudomallei*), and an ammonia-oxidizing archaeon (*Nitrosopumilus maritimus*), and an unrooted phylogenetic tree was constructed using the neighbor-joining algorithm. As shown in supplemental Fig. 1, the sequence of the *N. oceani* enzyme is highly homologous to that of the ‘class 1’ nitrite reductase from denitrifying bacteria, which is consistent with the patterns of insertion and deletion in the amino acid sequence (6). In contrast, all known enzymes from β-AOB, including *N. europaea*, were closely related with the ‘class 2’ enzymes found in non-denitrifying pathogenic bacteria, denitrifying archaea, and fungi. The phylogenetic position of *N. oceani* nitrite reductase suggests that lateral gene transfer between denitrifying bacteria and γ-AOB is more plausible than molecular evolution and divergence of the enzyme from the common ancestral enzyme during the evolution of β- and γ-AOBs.

**Fig. 2:**
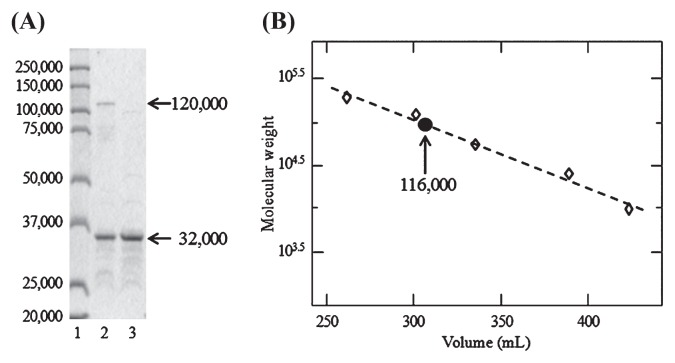
Determination of subunit composition of recombinant nitrite reductase. (A) SDS-PAGE analysis of the purified preparation that was pretreated by 2% SDS (lane 2) or by 2% SDS and 2% 2- mercaptoethanol (lane 3). Standard proteins are shown in lane 1. (B) The molecular weight of the recombinant protein in the solution was determined by gel filtration. Bovine liver catalase, alcohol dehydrogenase, hemoglobin, carbonic anhydrase, and horse mitochondrial cytochrome *c* were used as the standards.

The precursor protein of nitrite reductase from *N. oceani* was expressed in host *E. coli* BL21 cells; however, all the recombinant protein was obtained as inclusion bodies in the insoluble preparation. Attempts at solubilization and refolding of the recombinant protein did not succeed. Next, we amplified the gene without the 60-bp 5′-terminal nucleotides that correspond to the putative signal sequence. The amplicon was inserted into the expression vector, then the pETNc- NirKm construct was transduced into the host cells for expression of the recombinant protein without the signal sequence. A small part of the recombinant protein was obtained as a soluble form, but it seemed to be an apoprotein because it lacked nitrite reducing activity. The recombinant nitrite reductase was successfully activated by incorporation of Cu ions, then the holoenzyme obtained was purified to an electrophoretically homogeneous state by three chromatography steps, as summarized in Table 1 and Fig. 2A. The N-terminal of the recombinant protein was identical to the expected sequence, Met-Ala-Asp-Gly-Glu, containing an artificial N-terminal methionine and subsequent residues from the 21^st^ to 24^th^ amino acids of the *nirK* gene product. The molecular weight of the subunit molecule estimated by SDS-PAGE was lower than the value of 38,000 calculated from the DNA sequence, but the reason for this discrepancy is unclear.

When the purified preparation was denatured without being treated by 2-mercaptoethanol for SDS-PAGE analysis, a new band appeared on the gel at the position corresponding to the molecular weight of 120,000 (Fig. 2A). In addition, the molecular weight of the recombinant enzyme in the solution was estimated to be 116,000 by gel filtration (Fig. 2B). These experimental results and sequence information imply that the nitrite reductase was composed of three identical subunits whose molecular weight was 38,000 each. A homotrimeric triangular structure is a general characteristic of Cu-containing nitrite reductase and has been resolved by X-ray diffraction analysis of the crystal of the enzymes from denitrifying bacteria (12).

**Fig. 3:**
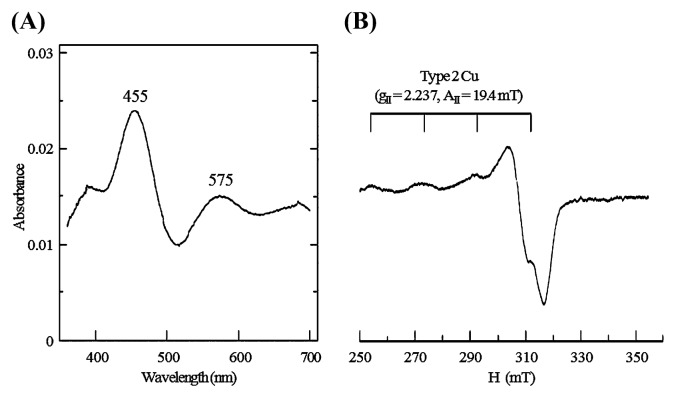
Absorption and EPR spectra of recombinant nitrite reductase. (A) The purified enzyme (0.368 mg protein mL^−1^) was dissolved in buffer A, and its absorption spectrum in the visible region was measured. (B) The enzyme (25.1 mg protein mL^−1^) was dissolved in buffer A containing 50% (v/v) glycerol, and the EPR spectrum was measured at 77 K. Conditions of the EPR run were microwave frequency, 8.85 GHz; microwave power, 7.00 mW; modulation amplitude, 100 KHz; sweep time, 8 min; time constant, 0.1 s.

The recombinant enzyme was green in the oxidized state and showed absorption peaks at 455 nm and 575 nm in the spectrum of the visible region, as shown in Fig. 3A. In contrast to the *N. europaea* enzyme, which was blue, showing a single absorption peak at 607 nm (8), the absorption spectrum of the NS58 enzyme resembled those of green-type nitrite reductases from denitrifying bacteria (19, 23). The millimolar extinction coefficient (ɛ_mM_) at 455 nm of the *N. oceani* enzyme was estimated to be 7.2 mM^−1^ cm^−1^ per enzyme molecule. The ɛ_mM_ value was comparable to those of green-type enzymes from *A. faecalis* S-6 (6.98 mM^−1^ cm^−1^ at 457 nm) (19) and *A. cycloclastes* (6.3 mM^−1^ cm^−1^ at 464 nm) (23). The concentration of Cu in the purified enzyme was determined to be 1.62±0.15 (mol per mol of the subunit molecule) as the mean value±standard deviation using four individual samples. This value is close to the expected stoichiometric value of 2 for the typical Cu-containing nitrite reductase involving two copper centers, type 1 (T1) Cu and type 2 (T2) Cu. These results suggested that the incorporation of Cu into the apoprotein was almost complete. Absorbance in the visible region was due to a T1 Cu, which mediates intramolecular electron transfer from a physiological electron donor at the reaction site to the catalytic T2 Cu for nitrite reduction in the enzyme. Comparison of the X-ray crystal structures indicated that only a slight difference in the spatial orientations of the Met side chain with T1 Cu produced a difference in the visible spectra between blue and green enzymes (15). The T2 Cu for the nitrite-reducing center of the enzyme has no absorption in the visible region, but shows characteristic EPR signals (23). As shown in Fig. 3B, hyperfine-splitting signals (g_II_=2.2375, A_II_=19.4 mT) in the EPR spectrum indicated the presence of the T2 Cu center in the recombinant. The EPR parameter was consistent with that of the green-type enzyme from *A. cycloclastes* (23). An EPR signal corresponding to the T1 Cu center was not observed. In the *Pseudomonas aureofaciens* nitrite reductase, the T1 Cu center has been reported to be rather labile and easily convertible to a Cu center with T2 properties without loss of nitrite reducing activity (38). Absence of a T1 Cu signal might be explained by the easy convertibility of the Cu center in *N. oceani* nitrite reductase.

Using reduced MV as an electron donor, the nitrite reducing activity of the purified recombinant enzyme was optimal at pH 5.5, and 50% of the above maximum activity was observed in a wide pH range from 5.0 to 7.0. The molecular activity (*k*_cat_) per subunit molecule and affinity constant (K_m_) for nitrite were determined to be 1.6×10^3^ s^−1^ and 52 μM, respectively, at the optimal pH by Hanes-Woolf plotting analysis. The catalytic efficiency (*k*_cat_/K_m_), which was calculated as 3.1×10^7^ M^−1^ s^−1^, is close to that of diffusion-controlled enzymes (10^8^–10^9^ M^−1^ s^−1^), indicating that the nitrite reduction catalyzed by the *N. oceani* enzyme is highly efficient. *N. oceani* enzyme activity was 180 times that of the specific activity (27 s^−1^) reported for the *N. europaea* enzyme when using reduced *N. europaea* cytochrome *c*552 as the reductant (8). The K_m_ value for the nitrite of the *N. europaea* enzyme has not been reported. The molecular and catalytic properties of the *N. oceani* enzyme were compared with those of enzymes from several kinds of organisms, as summarized in Table 2.

The physiological electron-donating component of the *N. oceani* nitrite reductase could not be defined. It has been generally accepted that blue-type nitrite reductase reacts with azurin and/or cytochrome *c* as the physiological electron donor, whereas the green enzyme reacts with pseudoazurin (20). A putative blue copper protein, encoded in the noc_0263 gene in the *N. oceani* ATCC19707 genome, revealed significant sequence similarity with pseudoazurin and may function as an electron donor for the present enzyme.

In this study, we prepared Cu-containing nitrite reductase from marine γ-AOB *N. oceani* as a recombinant protein, and its molecular and enzymatic properties were investigated. The enzymatic capacity of *N. oceani* nitrite reductase suggests that the enzyme can effectively remove intracellular nitrite to prevent its toxicity in cooperation with a putative NO reductase. Although catalytic activity of the nitrifier denitrification pathway in *N. oceani* has been reported (5), nitrite reducing activity was not detected in cultivated NS58 cells and the enzyme could not be purified in the mature state because of the low expression level of nitrite reductase in *N. oceani*. The *nirK* gene of denitrifying bacteria is up-regulated under anaerobic conditions (36). In contrast, expression of the *nirK* gene of *N. europaea* is not controlled by the redox level, but is regulated by the nitrite-dependent repressor NsrR (4). Jason *et al.* (18) have indicated the lack of a putative NsrR-binding motif in the promoter sequence of the *nirK* gene of *N. oceani* ATCC19707. Transcriptional regulation of the *nirK* gene in *N. oceani* is therefore also interesting and should be investigated in the future.

## Supplementary Material



## Figures and Tables

**Fig. 1:**
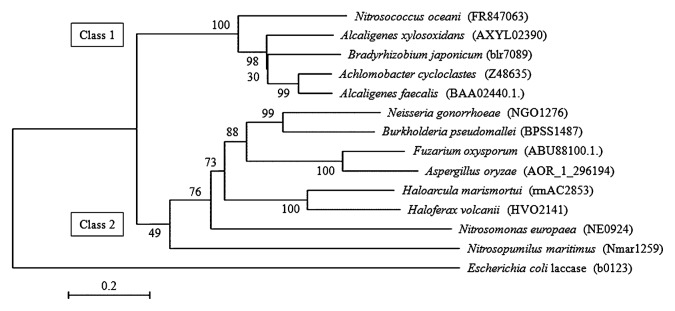
Phylogenetic relationships of Cu-containing nitrite reductases. The structure of the unrooted neighbor-joining tree is inferred from the alignment of the putative amino acid sequences of the nitrite reductases from denitrifying bacteria, *A. xylosoxidans*, *B. japonicum*, *A. cycloclastes* and *A. faecalis*; two non-denitrifying pathogenic bacteria *N. gonorrhoeae* and *B. pseudomallei*; two denitrifying fungi *F. oxysporum* and *A. oryzae*; two denitrifying archaea *H. marismortui and H. volcanii*; ammonia-oxidizing archaeon *N. maritimus*; β-AOB *N. europaea*; and marine γ-AOB *N. oceani*. Accession numbers of the nucleotide sequences of the *nirK* genes are indicated in parentheses. Bootstrap values derived from 1000 replicates are shown at the interior branches. *E. coli* laccase was used as the out-group. Scale bar represents 0.2 changes per sequence position.

**Table 1:** Purification of recombinant NirK from *N. oceani* NS58

Purification step	Volume	Total protein	Total activity	Specific activity	Yield

	(mL)	(mg)	(mmol of NO_2_^−^ min^−1^)	(μmol of NO_2_^−^ min^−1^ mg^−1^)	(%)
Insoluble fraction	20.0	426	n.d.	n.d.	—
Soluble fraction	40.0	346	n.d.	n.d.	—
Dialyzation	80.0	203	5.93	29.2	100
Anion exchange	21.0	24.4	2.93	120	49.3
Gel-filtration	21.0	8.79	2.95	336	49.8
Hydrophobic	8.0	1.23	2.67	2,181	45.1

n.d., not determined.

**Table 2:** Molecular and enzymatic properties of Cu-containing nitrite reductase

		Subunit composition^a^	Visible absorption peaks^b^ (nm)	EPR				Physiological electron donor	Activity^c^		Ref.
	
				T1 Cu		T2 Cu			Affinity constant for NO_2_^−^	Turnover	
		
				g_II_	A_II_ (mT)	g_II_	A_II_ (mT)		(μM)	(×10^3^ s^−1^)	
Class I	*N. oceani*	(38,000)×3	**455**, 575	—	—	2.237	19.4	—	52	1.600	Present study
	*A. cycloclastes*	(37,000)×3	**464**, 590	2.195	7.30	2.262	17.5	pseudoazurin	500	0.172	(12, 16, 17, 20, 23)
	*A. xylosoxidans*	(36,500)×3	460, **593**	2.208	6.30	2.298	14.2	azurin, cytochrome *c*553	34	0.445	(1, 9, 10, 24)

Class II	*N. europaea*	(40,100)×3	**607**	2.250	6.80	2.260	17.0	cytochrome *c*552	n.d.	0.027	(8)
	*N. gonorrheae*^d^	(36,200)×3	458, **585**	n.d.	n.d.	n.d.	n.d.	azurin	n.d.	0.290	(6)
	*H. marismortui*	(34,100 or 35,800)×3	465, **600**	2.232	4.40	2.304	13.3	—	n.d.	1.680	(14)
	*F. oxysporum*	(41,800)×2	470, **595**	2.220	6.82	2.320	n.d.	azurin, cytochrome *c*549	n.d.	0.621	(22)

a Molecular weight was calculated based on the nucleotide sequence.

b Maximum peaks are shown in bold.

c An artificial electron donor (reduced methylviologen or phenazine methosulfate) was used as the electron donor except for the *A. cycloclastes* enzyme (pseudoazurin) and *N. europaea* enzyme (cytochrome *c*552).

d Recombinant of the soluble domain.

n.d., not determined.
