# Supplementary material for: Expression, and Molecular and Enzymatic Characterization of Cu-Containing Nitrite Reductase from a Marine Ammonia-Oxidizing Gammaproteobacterium, Nitrosococcus oceani
Source: Microbes Environ. 2012 Apr 28;27(4):407–12. doi: 10.1264/jsme2.ME11310 (PMC4103548; doi:10.1264/jsme2.ME11310)
Supplement: Supplementary file 1 [file 27_407_s1.pdf]

Supplemental Fig. 1. Sequence alignment of Cu-containing nitrite reductases. Amino acid sequence of the enzyme from *N. oceani* (Nco) is aligned with those from *A. xylosoxidans* (Axy), *B. japonicum* (Bja), *A. cycloclastes* (Acy), *A. faecalis* (Afa), *N. gonorrhoeae* (Ngo), *B. pseudomallei* (Bps), *F. oxysporum* (Fox), *A. oryzae* (Aor), *H. marismortui* (Hma), *H. volcanii* (Hvo), *N. europaea* (Neu), and *N. maritimus* (Nma). Amino acids are numbered at the right margin. Putative Cu-binding residues for type 1 and type 2 Cu are indicated by I and II, respectively. Insertions and deletions of the sequence that are common in the class 1 or class 2 enzymes are boxed. Accession numbers of the sequences are indicated in Figure 1.

Supplemental Figure 1_1

```
Noc   ------------------------------------------------------MKKLIKYFAFYLATF    15
Axy   -----------------------------------------------------------------MNAL     4
Bja   --------------------------------------------------------------MLPMFTR     7
Acy   ---------------------------------------------MTEQLQMTRRTMLAGAALAGAVAP    24
Afa   ---------------------------------------------MAEQMQISRRTILAGAALAGALAP    24
Ngo   --------------------------------------------------MKRQALAAMIASLFALAAC    19
Bps   -------------------------------------------------MKDLQSIRGQLRATFLSVLL    20
Fox   MSRMVTICWTLAPATSQLLPIASRAAASTAFIRNLSLPTRRAPVIGARSFSSNTRSNSRVSHPRLTPALA    70
Aor   ----MNTAFSSLCRTKSRYASVTTIRGARRLPPRLLSSPCSPLRGPFLARQISTESRKGTSGFQIKYVLL    66
Hma   ---------------------------------------------------MSTIPTATRRRVLEALG    17
Hvo   ------------------------------------------------------MLSTTRRRTLQWLG    14
Neu   ---------------------------------------------------------MKYKQLLRGML    11
Nma   -----------------------------------------------MNKRVSMLFTIAAVAVMGATLF    22

Noc   SSVLYADGEASSYIANIGTHANLKDLERVR-----------------------------------------    45
Axy   RPTLLAMALIATMAGGPAAAQNADQLERAK-----------------------------------------    34
Bja   RAALISAAATALMLATPALAADDLKLPRQK-----------------------------------------    37
Acy   LLHTAQAHAAGAAAAAGAAPVDISTLPRVK-----------------------------------------    54
Afa   VLATTSAWGQGAVRKATAAEIAALPRQK-------------------------------------------    52
Ngo   GGEPAAQAPAETPAASAEAASSAAQATAETP----------------------------------------    50
Bps   GAVIAASAQAGAATGKVPGDFG-------------------------------------------------    42
Fox   VSTGLALSYYLYQRGPQPVKLGLAPEKPEKPKCAHCVDEKPEVIQEKAVGCCEDEQTTTYQEKPLSCCVD   140
Aor   LSTAATLSWLLYARQHGAVWLDSDKSYFTHPDPLAPNNGIHQQKTTNEIQDSQKETFKS-----------   125
Hma   VGTAALAGCASAPGAKEQATEAETTPQEPAM----------------------------------------    48
Hvo   LGGVASLAGCATKSPTAAQSLDETEEPTQ------------------------------------------    43
Neu   ASGFLLMG---------------------------------------------------------------    19
Nma   GSTYTQTQIAGQSLDMDQMDVSVMDKIRHMG----------------------------------------    53

Noc   ----------------------------------QELVAPPHLPDHQQAYSGKPRIVEIEMVIEEKEVE    80
Axy   ----------------------------------VALVAPPMVHPHEQVARSGPKVIEFTMTIEEKKMV    69
Bja   ----------------------------------VELVAPPFVHAHEQATKQGPKIVEFKLTIEEKKVV    72
Acy   ----------------------------------VDLVKPPFVHAHDQVAKTGPRVVEFTMTIEEKKLV    89
Afa   ----------------------------------VELVDPPFVHAHSQVAEGGPKVVEFTMVIEEKKIV    87
Ngo   ----------------------------AGELPVIDAVTTHAPEVPPAIDRDYPAKVRVKMETVE-KTM    90
Bps   ----------------------------PPQGEPLHATLVSPPNVPPPITRRYPAKVIVDLEVVE-KVL    82
Fox   DEASIAMVPAIGGDHKHKIFETVDDETLLISKLPKEDAILTTAPNVPPSITRDHPALVRVPLVTTT-KLA   209
Aor   --------------------------LEAEDLPTEKAILTTAPNVPPPITRDYPVILDVDLTAVA-KLE   167
Hma   ----------------------------NAAQQTDVDRIAADPTAIPDPIDRSEPKTVSVEMTTKE-QVA    89
Hvo   ----------------------------QGSTNLVDQVAADPTDIPGPIDRSESAEVDVTLRPEE-VTA    83
Neu   -------------------------------------------------IQAEAKTVQVTLHAVETDVA    39
Nma   ----------------------------------GLQLVMPEAFAETDCGALENSGRNVVEFNLTGESVEL    90

                                                 I     II
Noc   IEPN-VFVQAMTFNGTNPGPIIVVHEGDYVELTLKNLDTNMLLHNIDFHPATGALGGGALTKVAPGQQVT   149
Axy   IDDKGTTLQAMTFNGSMPGPTLVVHEGDYVELTLVNPATNAMPHNVDFHAATGALGGAKLTNVNPGEQAT   139
Bja   IDEKGTTFQAMTFNGSMPGPLMVVHEGDYVETTLVNPATNTMPHNIDFHSATGALGGGALTLINPGEQVV   142
Acy   IDREGTEIHAMTFNGSVPGPLMVVHENDYVER-LINPDTNTLLHNIDFHAATGALGGGALTQVNPGEETT   158
Afa   IDDAGTEVHAMAFNGTVPGPLMVVHQDDYLELTLINPETNTLMHNIDFHAATGALGGGGLTEINPGEKTI   157
Ngo   KMDDGVEYRYWTFDGDVPGRMIRVREGDTVEVEFSNNPSSTVPHNVDFHAATGQGGGAAATFTAPGRTST   160
Bps   PIADGVNYTFWTFGGAVPGNFIRVRQGDTVEFHLKNRPDSKMPHNIDLHAVTGPGGGATSSFTAPGHESR   152
Fox   QLTSQYKYEQWTFNGTVPGPFIRAKVGDVVELSLTNKDPAGNPHNIDCHAFTGPGGGAAVTTAEEGETKV   279
Aor   QLTNQYKYEKWTFNNSVPGPFIRARVGDIVNLKITNHDESGMPHNIDCHAFLGPGGGSALTTVNEGETKT   237
Hma   EIEPGVTYTYMTFGDQIPGPMIRVRRGDTVELTITNEEGNSMPHNIDLHAVRGPGGGAEASMVTPGQTKT   159
Hvo   EVEDGVTFTYTTYNGQVPGPLVRVRQGDTVNVTFENPEENTMPHNVDFHAVAGPGGGAEATMTNPGETAH   153
Neu   YDNKGSTYRAWTFDGKVPGPVVRVTEGDTVEFTLINDKNSKNSHSMDFHAAR-LDVVEDFESIKPGETKK   108
Nma   PIMGGKTYHAMTFSEQVPGPTLRVTQGDVVKMTLTIPDDEVTGHGNDMHASQMSAGN--FESVNPGETSQ   158
```

(continue)

Supplemental Figure 1_2

```
                         II I         I     I
Noc         LRFKADKAGTYVVYHCAPGGL-MTPYHVVSGMYGAIMIFPKKGLRDQNGKKVTYDKAYYVGEQGWYIPKDK   218
Axy         LRFKADRSGTFVVYHCAPEG--MVPWHVVAGMSGTLMVLPRDGLKDPQGKPLRYDRAYTIGEFDLYIPKDA   207
Bja         LRWKATKTGVFVYHCAPGGP-MIPWHVVSGMNGAVMVLPRDGLNDGKGHALKYDKVYYVGEQDMYVPRDE   211
Acy         LRFKATKPGVFVYHCAPEG--MVPWHVTSGMNGAIMVLPRDGLKDEKGQPLTYDKIYYVGEQDFYVPKDE   226
Afa         LRFKATKPGVFVYHCAPPG--MVPWHVVSGMNGAIMVLPREGLHDGKGKALTYDKIYYVGEQDFYVPRDE   225
Ngo         FSFKALQPGLYIYHCAVAP---VGMHIANGMYGLILVEPK-------EGLPKVDKEFYIVQGDFYTK---   217
Bps         FTFKALNEGLFVYHCATAP---VGMHVANGMYGLILVEPP-------EGLPKVDREYYVMQGDFYTN---   209
Fox         GRFKLLYPGLYVYHCAAAP---VPVHIANGMYGLMYVQPEG------NDLPPVDKEYYVMQSEFYHEPPE   340
Aor         ARFRLQNPGLYIYHCAVGP---VGVHIANGMYGLLYVQPE-------QDLPPVDKEYYVMQSEFYHEPPE   297
Hma         FRFKATYPGAFIYHCAVPN---LDMHISSGMFGMILVEPK-------EGLPEVDHEFYFGQHELYTT---   216
Hvo         LRFKATYPGAYIYHCAVPN---MDMHISAGMFGLILVEPP-------EGMPEVDHEIYLGQHELYTD---   210
Neu         YTFTADNPGVFFYHCGSDP---MIQHIARGMYGVIIVDPKD-----ANALPKADREYVLIQAEHYEN---   167
Nma         YCYIAESAGMFKYHCSGVKLIGMDQHVLSGMYGITIVDPANG--YKKLMVEKTSGSGELDRKFYDADALE   226

Noc         NGKYKRYANAIEPYGDTLEVMRGLVPTHVTYNGSKGALTG------------------------------   258
Axy         NGKYKDYPTLAESYGDTVAVMRTLTPSHIVFNGKVGALTG------------------------------   247
Bja         KGNFKSYDSPGEAFTDTEEMMKKLIPSHVVFNGKVGALTG------------------------------   251
Acy         AGNYKKYETPGEAYEDAVKAMRTLTPTHIVFNGAVGALTG------------------------------   266
Afa         NGKYKKYEAPGDAYEDTVKVMRTLTPTHVVFNGAVGALTG------------------------------   265
Ngo         -----GKKGAQGLQPFDMDKAVAEQPEYVVFNGHVGAIAG------------------------------   252
Bps         -----GKYREKGLQSFDMDKAIDERPTYVVFNGAEGALTG------------------------------   244
Fox         ---VDDDGRRSEIVEFSYPNGLREEPQVVAFNGSESALTR------------------------------   377
Aor         ---PDDNGQMSSTVEFSWPHALREAADVVVFNGSEAALT-------------------------------   333
Hma         -----GDTGEKGHHDFDMEAMAAEEPTYVLMNGEKYAITPDRH---------------------------   254
Hvo         -----KDAGEEGQHAFDYEAMRNEEPTYVLMNGEKYAWTPNGR---------------------------   248
Neu         --------------PDDKTAMMQNKWSNVVFNGGVFKYDPVHDS-----------------------EA   199
Nma         FQLQYNQLYLTPEGNYDAGAMFKHQNTATVVNGMQFGYVPNMAHNLLVNGDVNKNIFVAQPWNGLEHKQY   296

Noc         DNAMKAKVGDSVLFIHS--QANEDSRIHLIGGHGDLVWPGGSFKNTPMVDKETWHVNGGESVAALYTFKQ   326
Axy         ANALTAKVGETVLLIHS--QANRDTRPHLIGGHGDWVWETGKFANPPQKDLETWFIRGGSAGAALYTFKQ   315
Bja         KNALTANVGENVLIVHS--QANRDSRPHLIGGHGDYVWETGKFGNAPEVGLETWFIRGGSAGAAMYKFMQ   319
Acy         DHALTAAVGERVLVVHS--QANRDTRPHLIGGHGDYVWATGKFRNPPDLDQETWLIPGGTAGAAFYTFRQ   334
Afa         DKAMTAAVGEKVLIVHS--QANRDTRPHLIGGHGDYVWATGKFNTPPDVDQETWFIPGGAAGAAFYTFQQ   333
Ngo         DNALKAKAGETVRMYVGNGGPNLVSSFHVIGEIFDKVYVEGGKLINEN--VQSTIVPAGGSAIVEFKVDI   320
Bps         ERAMRARTDETVRLFVGNGGPNLVSSFHVIGAVFDKVRADGSNVTQND--VQTTLIPAGGAATIEFHTRV   312
Fox         DHPLKAHVGDDVRIFFGNAGPNLTSSFHIIGTHFKNVYRDGGVTSNPSKGIQTVSVPCGGSTIVDLKMAV   447
Aor         EKPLKATLDDTVRIFFGNGGPNLTSSFHVIGTCFNKVYRDSDVLSPPGQCVQTVSVPPGGSTIVDMKMVV   403
Hma         G-SPSMQVGETARVYFVTGGPNLDSSFHPIGSVWDEVWQQGSIAGPPNRYVQTTPVKPGSCAIATLHAEV   323
Hvo         GPAATVGLDETVRVYFVDGGPNLSSSFHPIGSVWETLYPEGSLTTEPQTHIQTRQVPPGSTTIATMSSPV   318
Neu         TSWLQAKPGERVRIYFVNAGPNELSSLHPIAGIWDRVYPSGN-PKNVQYALQSYLIGAGDAATLDLISPV   268
Nma         QSQLLFVENDQHVRLFVENQGNEPVFFHIVGEILDRVTQGNRVQSAAT---ETWLLGGSQGMIVDLVFDE   363
                    II
Noc         PGLYAYLNHNLIQAFMLGGAAHVKVDGE-WNNDLMEQVVKPSPINAD.                         372
Axy         PGVYAYLNHNLIEAFELGAAGHIKVEGK-WNDDLMKQIKAPGPIPR.                          360
Bja         PGIYAYVTHNLIEAADLGATAHFKVEGK-WNDDLMTQVKAPAEIPANTN.                       367
Acy         PGVYAYVNHNLIEAFELGAAGHFKVTGE-WNDDLMTSVVKPASM.                            377
Afa         PGIYAYVNHNLIEAFELGAAAHFKVTGE-WNDDLMTSVLAPSGT.                            376
Ngo         PGNYTLVDHSIFRAFNKGALGQLKVEGA-ENPEIMTQKLSDTAYAGSGAAS(+22aa).              392
Bps         PGNYTFVDHSIFRAFNKGALAILKVDGP-ENKAIYSGKELDAPYSG-DAVT(+159aa).             520
Fox         PGTYTLVDHSIFR-LDKGAVGFLNVSGP-QNPGVYQSSQPPRPCVGCKLHS.                     496
Aor         PGTYTIVDHAIFR-LEKGAKGFLNVSGE-PRPMLYYSTLPPQPCEGFNLKY(+107aa).             559
Hma         PGPIKLVDHALSRVARKGTMAIINREGA-ANPDVFEPEA.                                 361
Hvo         PGDFKLVDHSLSRVVRKGCMAVVRAEGA-EDPEIFDPDPDPQ.                              359
Neu         EGANAIVDHSMRHAHSGAIAVIMFTNDA-DPEAGRGENILIR.                              309
Nma         PGAYAAVNHDYAAIYTGAATVFVAGDPFGLNPVLVEKGVIPAPVASYAYAL(+56aa).              470
```
